# The origin of consciousness and beyond

**DOI:** 10.3389/fpsyg.2014.01385

**Published:** 2014-11-26

**Authors:** Andrea E. Cavanna

**Affiliations:** ^1^Michael Trimble Neuropsychiatry Research Group, University of Birmingham and BSMHFTBirmingham, UK; ^2^Department of Clinical and Systems Neuroscience, School of Life and Health Sciences, Aston UniversityBirmingham, UK; ^3^Sobell Department of Motor Neuroscience and Movement Disorders, UCL Institute of Neurology, University College LondonLondon, UK

**Keywords:** consciousness, evolution, self concept, epistemology, psychology

The origin of consciousness is one the most intractable mysteries about the human mind, because of its intrinsic conceptual and theoretical difficulties. By adopting a truly multidisciplinary approach, Graham Little tackles this problem in an original and accessible book. The author endorses the view that consciousness can only be discussed from within a general theory of psychology. He therefore devotes the first part of his book to the development of a sound methodology which can be used to create a psychological theory of the person. In doing so, he acknowledges the inspiring role of William Ross Ashby's work in cybernetics and analysis of self-correcting systems. The second part of the book explores the consequences of the psychological theory of the person to the multifaceted nature of humankind.

The human brain is defined as the highest state of evolved neural structure adapted to make maximum survival use of the environmental niche of differentiated perceptual fields. One of the key steps in the origin of consciousness was the development of the neural capacity to record and group events according to their properties, thereby generating ideas. All ideas are knowledge of past events (“borrowed knowledge” in Ashby's 1960, terminology), which can be linked in chains to describe and predict the flow of change in reality, based on historical experience. Therefore the potential for generating ideas provided our species with a definite survival advantage (“survival through knowledge”). An Ashby diagram is used to describe the structure of the human psyche with fully defined variables (environment, body, reacting part, brain structures, attitude, emotion, knowledge, and attention) and the links between them as indicative of the flow of change through the system “person in their environment.” The brain is understood as an entropic device whereby the energy inevitably flows to the lowest energy states/pathways available to it, i.e., those states that arose from historical experience (habits). Crucially, neural evolution also developed an attention mechanism alerting the individual to danger. The attention mechanism of the brain enabling redirection of neural flows contrary to entropy is called choice, or free will, and is the only system in the universe not directed by entropy. Creative free will is intrinsically unpredictable, and is the core inherent strength of humankind enabling it to overcome entropy by creating and adopting new ideas whenever the decision is made to invest the effort.

The “I,” i.e., the conceptualisation of the human spirit, is the core of the psyche, and as such holds a special and central place in our self-awareness. The remainder of the evolved structure of the psyche, including knowledge, emotions, and attitudes, is defined as mind. The attention mechanism of the brain and the sense of self, embedded in the “I,” combine to create human consciousness. Therefore, consciousness is a function of the content of the brain, not the mechanism of the brain. A fundamental aspect of human experience is the resolution of the tension between entropy and free will. The “I” learns that the attention mechanism can intervene in the brain to cause neural flows to take paths they would not otherwise take. The “I” can either intervene and redirect the flow of neural energy to thwart entropy (an option which requires energy and effort) or acquiesce to entropy, thereby allowing life to flow in the direction dictated by the past. The applications of this theory are far-reaching, as it can account for a range of aspects of human life, including intelligence, habit, choice, emotions, knowledge, nature/nurture, and mental health/illness. The themes covered by the book reflect the breadth of the theory's implications. A few examples include epistemology and the problem of knowledge (chapters 4, 10, and 34), perception (chapter 5), psychological theory (chapter 11), free will (chapter 16), dreaming (chapter 21), learning (chapter 22), intelligence (chapter 23), artificial intelligence (chapter 25), spirituality (chapter 26), mental illness (chapter 27), causality (chapter 30), and modern physics (chapter 33). In consideration of its breadth, this book embraces a wide readership, which includes, but is not restricted to, psychologists, social scientists, philosophers, and neuroscientists.

In the book's dense appendix, titled “Toward a better standard of judgment than peer review,” the author argues that the current standard of “peer review” for the evaluation of scientific papers should be replaced by “rigorous strategic and conceptual transparency.” This is based on the observation that the peer review process when empirically tested failed in protecting the system from the publication of articles of low and inadequate intellectual quality, as shown by Alan Sokal with his famous hoax paper (Sokal and Bricmont, 1998). The book closes with a short chapter outlining the author's intellectual development, along with the reason for the deliberate choice not to include a structured bibliography (“I disagree with a modern approach to scholarship whereby references or at least the volume of references, in some way infers intellectual standing. I believe we need return to full and vigorous assessment of ideas in their own right; it is the quality of the thinking and the integrity of the identification of source that marks the worth”).

Two late authors sprang to my mind when reading Graham Little's book on the origin of consciousness. The first one is American psychologist Julian Jaynes, who also adopted a multidisciplinary approach to the problem of the origin of consciousness in his 1976 book “*The Origin of Consciousness in the Breakdown of the Bicameral Mind*” (Jaynes, 1976), albeit with more focus on neurological and archeological findings (Cavanna et al., 2007). The second author is Austrian philosopher Paul Feyerabend, who since the publication of his book “*Against Method*” (Feyerabend, 1975) became of one the modern champions of the critical approach to both scientific method and research practice. Interestingly, Feyerabend's background also touched on philosophy of mind and consciousness studies (Feyerabend, 1963). Does Graham Little's thought-provoking book provide the ultimate answer to the question of the origin of consciousness? Readers will find their answers at the end of this intellectual journey through what comes across as an interesting and original attempt from a genuinely free thinker.

## Conflict of interest statement

The author declares that the research was conducted in the absence of any commercial or financial relationships that could be construed as a potential conflict of interest.
